# High Genetic Diversity of Carbapenem-Resistant Acinetobacter baumannii Isolates Recovered in Nigerian Hospitals in 2016 to 2020

**DOI:** 10.1128/msphere.00098-23

**Published:** 2023-04-17

**Authors:** Erkison Ewomazino Odih, Anderson O. Oaikhena, Anthony Underwood, Yaovi Mahuton Gildas Hounmanou, Oyinlola O. Oduyebo, Abayomi Fadeyi, Aaron O. Aboderin, Veronica O. Ogunleye, Silvia Argimón, Vitus Nnaemeka Akpunonu, Phillip O. Oshun, Abiodun Egwuenu, Tochi J. Okwor, Chikwe Ihekweazu, David M. Aanensen, Anders Dalsgaard, Iruka N. Okeke

**Affiliations:** a Global Health Research Unit for the Genomic Surveillance of Antimicrobial Resistance, Department of Pharmaceutical Microbiology, Faculty of Pharmacy, University of Ibadan, Ibadan, Oyo State, Nigeria; b Department of Veterinary and Animal Sciences, Faculty of Health and Medical Sciences, University of Copenhagen, Copenhagen, Denmark; c Centre for Genomic pathogen Surveillance, Big Data Institute, University of Oxford, Oxford, United Kingdom; d Department of Medical Microbiology and Parasitology, Faculty of Basic Medical Sciences, College of Medicine, University of Lagos, Lagos, Nigeria; e Department of Medical Microbiology and Parasitology, University of Ilorin, Ilorin, Kwara State, Nigeria; f Department of Medical Microbiology and Parasitology, Obafemi Awolowo University Teaching Hospitals Complex, Ile-Ife, Nigeria; g Department of Medical Microbiology and Parasitology, University College Hospital, Ibadan, Oyo State, Nigeria; h Clina-Lancet Laboratories, Victoria Island, Lagos State, Nigeria; i Nigeria Centre for Disease Control, Abuja, Nigeria; JMI Laboratories

**Keywords:** *Acinetobacter baumannii*, antimicrobial resistance, *bla*_NDM-1_, *bla*_OXA-23_, carbapenem resistance, genomics, surveillance

## Abstract

Acinetobacter baumannii causes difficult-to-treat infections mostly among immunocompromised patients. Clinically relevant A. baumannii lineages and their carbapenem resistance mechanisms are sparsely described in Nigeria. This study aimed to characterize the diversity and genetic mechanisms of carbapenem resistance among A. baumannii strains isolated from hospitals in southwestern Nigeria. We sequenced the genomes of all A. baumannii isolates submitted to Nigeria’s antimicrobial resistance surveillance reference laboratory between 2016 and 2020 on an Illumina platform and performed *in silico* genomic characterization. Selected strains were sequenced using the Oxford Nanopore technology to characterize the genetic context of carbapenem resistance genes. The 86 A. baumannii isolates were phylogenetically diverse and belonged to 35 distinct Oxford sequence types (^oxf^STs), 16 of which were novel, and 28 Institut Pasteur STs (^pas^STs). Thirty-eight (44.2%) isolates belonged to none of the known international clones (ICs). Over 50% of the isolates were phenotypically resistant to 10 of 12 tested antimicrobials. The majority (*n* = 54) of the isolates were carbapenem resistant, particularly the IC7 (^pas^ST25; 100%) and IC9 (^pas^ST85; >91.7%) strains. *bla*_OXA-23_ (34.9%) and *bla*_NDM-1_ (27.9%) were the most common carbapenem resistance genes detected. All *bla*_OXA-23_ genes were carried on Tn*2006* or Tn*2006*-like transposons. Our findings suggest that a 10-kb Tn*125* composite transposon is the primary means of *bla*_NDM-1_ dissemination. Our findings highlight an increase in *bla*_NDM-1_ prevalence and the widespread transposon-facilitated dissemination of carbapenemase genes in diverse A. baumannii lineages in southwestern Nigeria. We make the case for improving surveillance of these pathogens in Nigeria and other understudied settings.

**IMPORTANCE** Acinetobacter baumannii bacteria are increasingly clinically relevant due to their propensity to harbor genes conferring resistance to multiple antimicrobials, as well as their ability to persist and disseminate in hospital environments and cause difficult-to-treat nosocomial infections. Little is known about the molecular epidemiology and antimicrobial resistance profiles of these organisms in Nigeria, largely due to limited capacity for their isolation, identification, and antimicrobial susceptibility testing. Our study characterized the diversity and antimicrobial resistance profiles of clinical A. baumannii in southwestern Nigeria using whole-genome sequencing. We also identified the key genetic elements facilitating the dissemination of carbapenem resistance genes within this species. This study provides key insights into the clinical burden and population dynamics of A. baumannii in hospitals in Nigeria and highlights the importance of routine whole-genome sequencing-based surveillance of this and other previously understudied pathogens in Nigeria and other similar settings.

## INTRODUCTION

Antimicrobial resistance (AMR) is a global public health problem, with an estimated 1.27 million deaths globally attributable to drug-resistant bacteria in 2019 (1). This estimated AMR burden is substantially higher in low-resource settings, and carbapenem-resistant Acinetobacter baumannii represents one of the leading causes of deaths associated with or attributable to AMR (1).

Carbapenem resistance in A. baumannii is mediated primarily by carbapenemase enzymes, particularly those belonging to beta-lactamase class D (oxacillinases [OXA]) (2, 3). Oxacillinases, including OXA-23, OXA-24, and OXA-58, are common among A. baumannii strains and are each encoded by various alleles with various hydrolytic capacities. Although the intrinsic *oxaAb* gene encoding OXA-51 is harbored by all A. baumannii strains, only certain alleles of this gene may confer carbapenem resistance when overexpressed due to the presence of an upstream IS*Aba1* promoter (4, 5). OXA-23 is the most common acquired oxacillinase in A. baumannii, and it is typically flanked by insertion elements that facilitate its effective spread through conjugative plasmids across different A. baumannii lineages (6). The less common but significantly more potent (wider hydrolytic spectrum) New Delhi metallo-beta-lactamases (NDM), particularly NDM-1, have been reported more frequently in recent studies, including in Nigeria and other parts of Africa (7–10). Characterization of the genetic diversity and contexts of carbapenemase genes in various lineages would provide insights into the population dynamics of A. baumannii.

Two highly successful and widely disseminated A. baumannii lineages, international clone 1 (IC1) and IC2, predominate globally (11). In a review of all A. baumannii genomes present in the National Center for Biotechnology Information’s GenBank database in 2019, 61% were IC2, and 5% were IC1 (3). International clone 2 and, to a lesser extent, IC1 strains are also responsible for the majority of the carbapenem-resistant A. baumannii outbreaks reported in hospitals throughout the world (7, 12–14). However, as the success and global dissemination of these clones are associated with their propensity to carry multiple antimicrobial resistance determinants (13, 15), reports of increasing carbapenem resistance among A. baumannii isolates belonging to nonmajor global clones and the emergence and spread of previously uncharacterized highly resistant clones in different hospital settings are worrisome (16–18). This necessitates the characterization of the A. baumannii diversity and prevalent resistance mechanisms in different geographical contexts to inform future infection prevention and control measures and vaccination efforts. This study aimed to determine and characterize the lineages and carbapenem resistance mechanisms of A. baumannii isolates in southwestern Nigeria.

## RESULTS

### Isolate collection.

One hundred twenty-five isolates were submitted as presumptive A. baumannii to the reference laboratory during the study period. Of these, 100 isolates were confirmed as Acinetobacter species based on whole-genome sequencing (WGS) identification and included in the analyses. A further seven isolates originally submitted to the reference laboratory as Klebsiella pneumoniae (*n* = 3), Escherichia coli (*n* = 2), Enterobacter cloacae (*n* = 1), and Staphylococcus haemolyticus (*n* = 1) but determined to be misidentified and confirmed to be A. baumannii by WGS were also included. Thus, a total of 107 isolates, including 36 isolates characterized in our previous study (7), were included in the analyses. These comprised A. baumannii (86; 80.4%), Acinetobacter nosocomialis (16; 15.0%), Acinetobacter haemolyticus (two; 1.9%), Acinetobacter pittii (one; 0.9%), Acinetobacter indicus (one; 0.9%), and Acinetobacter variabilis (one; 0.9%). Specimen information was unavailable for 50 isolates. Of the remaining 57 isolates, 36 were isolated from blood (33 A. baumannii, two A. nosocomialis, and one *A. variabilis*), 20 isolates were from rectal swabs (19 A. baumannii and one A. pittii), and there was one A. baumannii isolate from cerebrospinal fluid.

### Diversity of clinical Acinetobacter baumannii strains in southwestern Nigeria.

**FIG 1:**
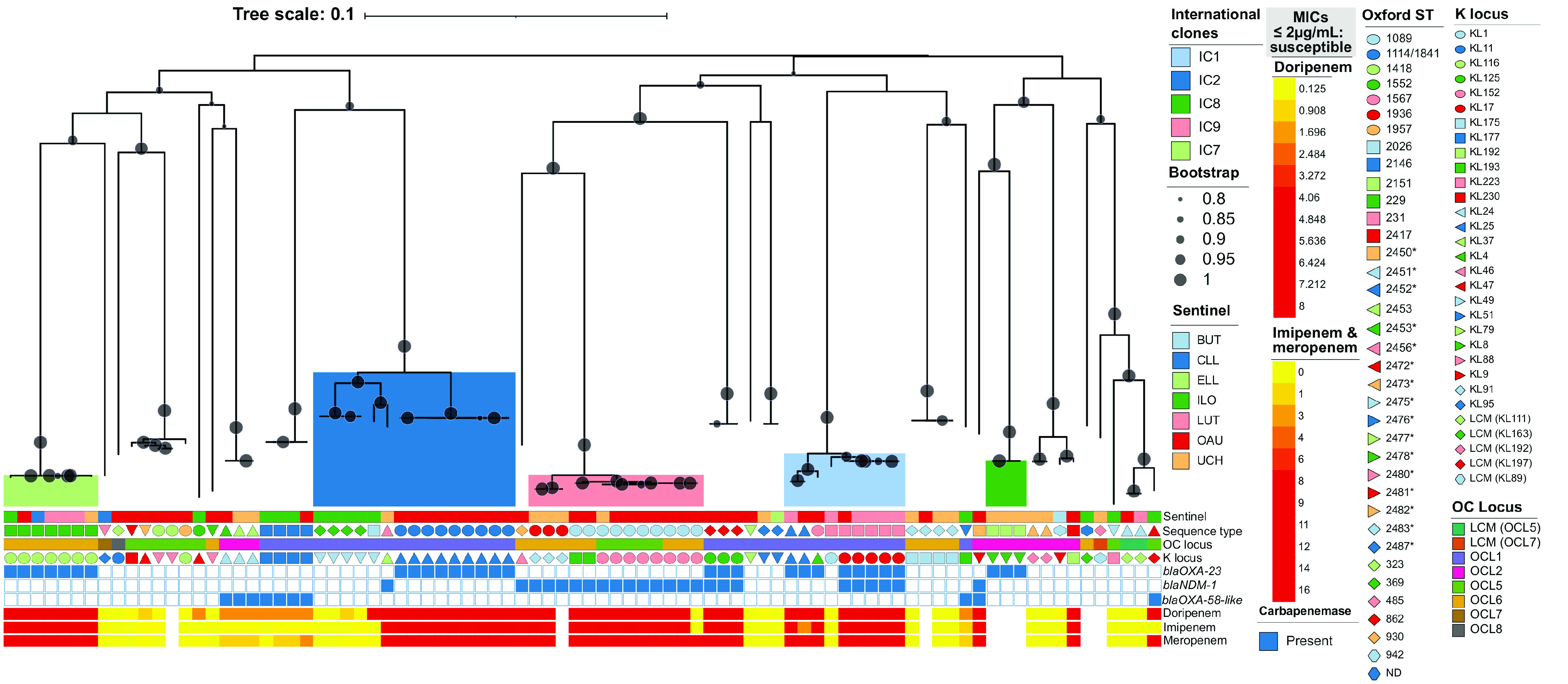
Maximum likelihood phylogeny of A. baumannii isolates. Colored clades represent international clones. BUT, Babcock University Teaching Hospital, Ilishan-Remo, Ogun State; CLL, Clina-Lancet Laboratories, Victoria Island, Lagos State; ELL, EL-LAB Medical Diagnostics, Festac, Lagos State; ILO, University of Ilorin Teaching Hospital, Ilorin, Kwara State; LUT, Lagos University Teaching Hospital, Idi-Araba, Lagos State; OAU, Obafemi Awolowo University Teaching Hospitals Complex, Ile-Ife, Osun State; UCH, University College Hospital, Ibadan, Oyo State.

The isolates belonged to 35 different Oxford sequence types (^oxf^ST), 16 of which were novel (*n* = 25; 29.1%), and 28 distinct Institut Pasteur STs (^pas^ST), seven of which were novel (see Fig. S1 in the supplemental material). The top 10 nonnovel sequence types detected included ^oxf^ST1089/^pas^ST85 (*n* = 10; 11.6%), ^oxf^ST1114/1841/^pas^ST2 (*n* = 9; 10.5%), ^oxf^ST229/^pas^ST25 (*n* = 7; 8.1%), ^oxf^ST231/^pas^ST1 (*n* = 6; 7.0%), ^oxf^ST2146/^pas^ST821 (*n* = 4; 4.7%), ^oxf^ST369/^pas^ST2 (*n* = 4; 4.7%), ^oxf^ST1936/^pas^ST85 (*n* = 3; 3.5%), ^oxf^ST2151/^pas^ST10 (*n* = 3; 3.5%), ^oxf^ST862/^pas^ST149 (*n* = 3; 3.5%), and ^oxf^ST1418/^pas^ST164 (*n* = 2; 2.3%). One isolate (^pas^ST203) could not be typed on the Oxford scheme as it was missing the *gdhB* locus. Nearly half (38; 44.2%) of the isolates either were singletons or belonged to none of the nine known ICs. The remaining isolates were IC2 (*n* = 15; 17.4%), IC9 (*n* = 13; 15.1%), IC1 (*n* = 9; 10.5%), IC7 (*n* = 7; 8.1%), or IC8 (*n* = 3; 3.5%). All identified ICs formed distinct phylogenetic clusters with no geographic clustering (Fig. 1). All the major lineages, except IC8 (^pas^ST10), which comprised only three isolates, were detected in at least two different locations. IC7 (^pas^ST25) was the only lineage detected in all five states sampled (five of the seven institutions).

10.1128/msphere.00098-23.1 FIG S1 Multilocus sequence types and international clone distribution of 86 A. baumannii isolates from southwestern Nigeria, 2016 to 2020. (A) Oxford sequence types. (B) Institut Pasteur sequence types. Download FIG S1, EPS file, 0.2 MB. Copyright © 2023 Odih et al. 2023 Odih et al. https://creativecommons.org/licenses/by/4.0/ This content is distributed under the terms of the Creative Commons Attribution 4.0 International license.

There was a high diversity with respect to the outer polysaccharide capsular loci (KL), with 31 distinct KL configurations detected among the isolates. The most common KL loci detected included KL25 (*n* = 11; 12.8%), KL116 (8; 9.3%), and KL152 (8; 9.3%). There was lesser diversity of the lipooligosaccharide outer core loci (OCL), but there was evidence of homologous recombination of the OCL between distantly related lineages. The majority of the isolates (35; 40.7%) had the OCL1 configuration. Twenty-two isolates (25.6%) had OCL6, 13 (15.1%) had OCL5, 11 (12.8%) had OCL2, and there was one isolate each with the OCL7 (1.2%) and OCL8 (1.2%) configuration. The remaining three isolates matched the OCL5 (*n* = 2; coverage, 80.77%; 7/9 expected genes present) and OCL7 (*n* = 1; coverage, 77.71%; 6/9 expected genes present) configurations most closely but with no match confidence.

### Antimicrobial resistance rates.

Rates of phenotypic resistance to all the tested antimicrobials were high among the A. baumannii isolates, with at least 50% resistance recorded for 10 of the 12 tested antimicrobials. Only minocycline (29.1%) and tigecycline (26.6%) had lower resistance rates. The resistance rates were, however, significantly higher among known international clone lineages than among the singletons/noninternational clones (adjusted *P* ≤ 0.009; Table 1). The IC7 (^pas^ST25) strains had the highest rates of resistance; all seven isolates (100%) were resistant to meropenem, imipenem, doripenem, and all the other tested antimicrobials except tigecycline (0%) and minocycline (0%). The IC9 (^pas^ST85) strains had similarly high rates of resistance to the carbapenems and other antimicrobials, as well as low rates of resistance to the tetracyclines (tigecycline, 25%; minocycline, 16.7%). Interestingly, the globally disseminated IC1 (^pas^ST1) and IC2 (^pas^ST2) strains had the highest rates of resistance to tigecycline (22.2% and 66.7%) but showed relatively lower resistance to the carbapenems than IC7 (^pas^ST25) and IC9 (^pas^ST85). Phenotypic susceptibility to colistin was also determined using the Vitek 2 system, but the results are not presented here as this is not the recommended method for correct determination of colistin resistance in A. baumannii.

**TABLE 1:** Antimicrobial resistance rates of clinical A. baumannii isolates from southwestern Nigeria, 2016 to 2020

Antimicrobial	Lineage (% resistance)					*P* ^*a*^	Adjusted *P*^*b*^
	IC1 (*n* = 9)	IC2 (*n* = 15)	IC7 (*n* = 7)	IC9 (*n* = 12)	Singleton/non-IC (*n* = 35)		
Cefepime	100	100	100	100	65.7	0.002	0.004
Ceftazidime	100	93.3	100	100	57.1	<0.001	0.001
Ciprofloxacin	100	93.3	100	100	65.7	0.009	0.009
Doripenem	88.9	73.3	100	91.7	45.7	0.002	0.004
Gentamicin	100	100	100	100	57.1	<0.001	<0.001
Imipenem	88.9	66.7	100	91.7	20	<0.001	<0.001
Levofloxacin	100	93.3	100	100	65.7	0.009	0.009
Meropenem	88.9	66.7	100	100	40	<0.001	<0.001
Minocycline	66.7	73.3	0	16.7	11.4	<0.001	<0.001
Piperacillin-tazobactam	100	100	100	100	60	<0.001	0.001
Ticarcillin-clavulanic acid	88.9	80	100	100	54.3	0.003	0.004
Tigecycline	22.2	66.7	0	25	17.1	0.003	0.004

a Fisher’s exact test comparison of resistance rates between the different lineage groups.

b Adjusted *P* values with false-discovery rate correction.

Although the noninternational clone isolates had significantly lower resistance rates than the isolates within the known ICs, they still showed at least 50% resistance to most of the tested antibiotics, including cefepime (65.7%), ciprofloxacin (65.7%), levofloxacin (65.7%), piperacillin-tazobactam (60%), ceftazidime (57.1%), gentamicin (57.1%), and ticarcillin-clavulanic acid (54.3%). Rates of resistance to the carbapenems were similarly lower among these strains but were nonetheless noteworthy (doripenem, 45.7%; meropenem, 40%; imipenem, 20%).

### Genotypic characterization of antimicrobial resistance.

**FIG 2:**
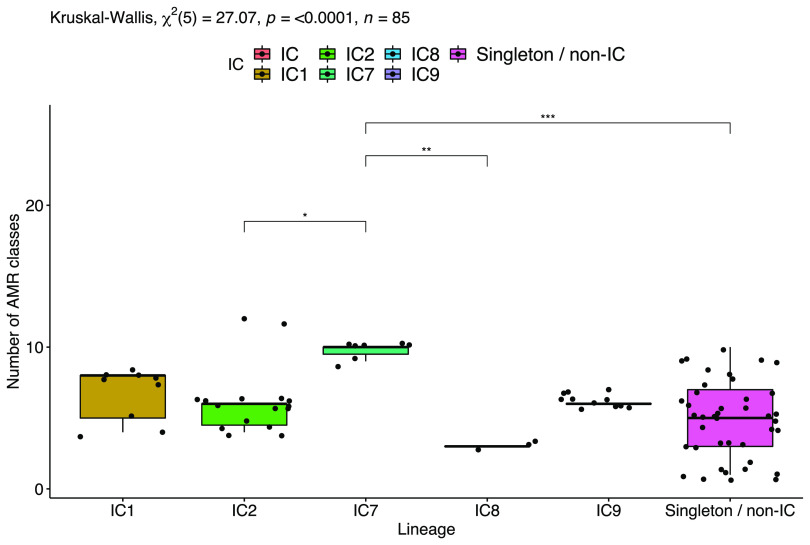
Comparison of the number of antimicrobial classes for which A. baumannii isolates in each lineage carried at least one resistance-conferring gene.

Similar to the phenotypic results, the number of antibiotic classes for which specific lineages carried at least one resistance-conferring gene was significantly different. The ^pas^ST25 (IC7) strains carried genes conferring resistance to significantly more antimicrobial classes compared with the ^pas^ST2 (IC2) (mean, 10 classes versus 7 classes; adjusted *P* = 0.01), ^pas^ST10 (IC8) (mean, 10 versus 3; *P* = 0.001), and non-IC/singleton (mean, 10 versus 5; *P* = 0.0001) strains (Fig. 2). Interestingly, however, with disaggregated data, a novel ST (^oxf^ST2456; ^pas^ST2) belonging to IC2 carried genes conferring resistance to the highest number of antimicrobial classes (median, 12 classes), followed by the ^pas^ST25 (IC7) strains (10 classes) and another novel ST (^oxf^ST2450; ^pas^ST1093) isolate (10 classes). In general, the A. baumannii isolates carried a higher number of genes conferring resistance to distinct antimicrobial classes (median = 6 classes) than did the non-*baumannii* isolates (*A. haemolyticus *= 4 classes, A. pittii = 3, A. nosocomialis = 2, *A. indicus *= 1, and *A. variabilis *= 1).

All 86 A. baumannii isolates (100%) carried at least one aminoglycoside resistance gene, including *aac(3)-Ia*, *aac(3)-IId*, *aac(3)-IIe*, *aac(6′)-Ian*, *aacA16*, *aadA1*, *aadA2*, *ant(2″)-Ia*, *ant(3″)-IIa*, *aph(3″)-Ib*, *aph(3′)-Ia*, *aph(3′)-Ib*, *aph(3′)-VI*, *aph(3′)-VIa*, *aph(6)-Id*, and *armA* (Table S2). Sulfonamide (*sul1* and/or *sul2*; *n* = 71; 82.6%) resistance genes and quinolone resistance-conferring mutations (*gyrA*_S81L, *parC*_E88K, *parC*_S84F, and/or *parC*_S84L; *n* = 70; 81.4%) were also present in a high proportion of isolates. Fifty-five isolates (64.0%) carried at least one carbapenem resistance gene, while 51 (59.3%) carried a tetracycline resistance gene [*tet(39)*, *tet(A)*, *tet(B)*, and/or *tet(G)*]. Some of the other common resistance genes detected include those conferring resistance to macrolides (*n* = 34; 39.5%), beta-lactams (*n* = 24; 27.9%), bleomycin (*n* = 24; 27.9%), trimethoprim (*n* = 16; 18.6%), and chloramphenicol (*n* = 13; 15.1%). No gene or mutation conferring colistin resistance was detected in the isolates in this study.

10.1128/msphere.00098-23.4 TABLE S2 Antimicrobial resistance genes detected in Acinetobacter species isolates in southwestern Nigeria, 2016 to 2020. Download Table S2, XLSX file, 0.01 MB. Copyright © 2023 Odih et al. 2023 Odih et al. https://creativecommons.org/licenses/by/4.0/ This content is distributed under the terms of the Creative Commons Attribution 4.0 International license.

Similar to the A. baumannii isolates, the aminoglycoside (*n* = 10, 47%) and sulfonamide (*n* = 9; 42.9%) resistance genes were the most abundant among the 21 non-*baumannii* isolates. Genes conferring resistance to tetracycline [*n* = 8; 38.1%; *tet(39)* and/or *tet(B)*] and carbapenem (*bla*_NDM-1_, *bla*_OXA-214_, and *bla*_OXA-420_ [*bla*_OXA-58_ family]; *n* = 7; 33.33%) were also present in at least a third of these isolates. The seven isolates carrying carbapenem resistance genes included the two *A. haemolyticus* isolates (both carried *bla*_OXA-214_) and five of the 16 A. nosocomialis isolates (all five carried *bla*_NDM-1_, and four cocarried the *bla*_OXA-58_-family *bla*_OXA-420_ gene).

### Distribution and genomic context of acquired carbapenem resistance genes in A. baumannii isolates.

**FIG 3:**
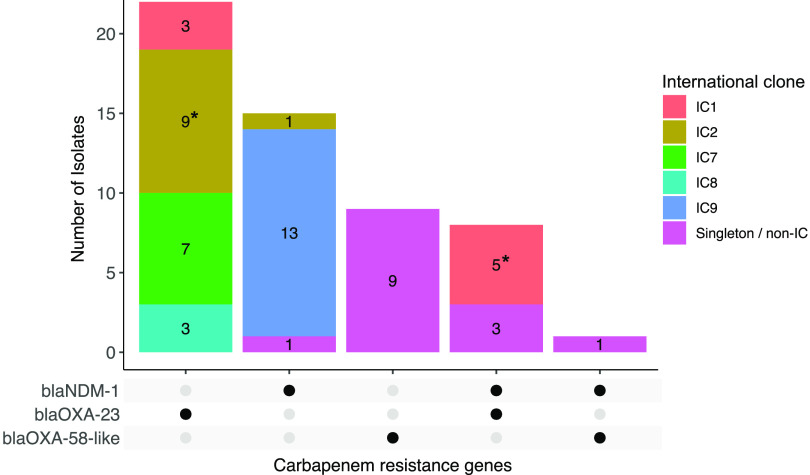
Lineage distribution and cocarriage of carbapenem resistance genes among A. baumannii isolates in southwestern Nigeria, 2016 to 2020. *, carried two copies of the *bla*_OXA-23_ gene.

The *bla*_OXA-23_ gene, present in 30 (34.9%) of the 86 A. baumannii isolates, was the most common acquired carbapenem resistance gene detected, followed closely by *bla*_NDM-1_ (*n* = 24; 27.9%). The only other carbapenem resistance genes detected among the isolates were the *bla*_OXA-58_-like genes (*bla*_OXA-58_ and *bla*_OXA-420_), which were present in 10 (11.6%) isolates. The *bla*_OXA-23_ genes were almost exclusive to the international clones, being carried predominantly by ^pas^ST1 (IC1; 8/9), ^pas^ST2 (IC2; 9/15), ^pas^ST25 (IC7; 7/7), and ^pas^ST10 (IC8; 3/3) isolates; only three non-IC isolates (^pas^ST149) harbored this gene (Fig. 3). These three isolates, as well as five of the IC1 isolates (^oxf^ST231), cocarried the *bla*_NDM-1_ gene in addition to *bla*_OXA-23_. Among the remaining six IC2 (^pas^ST2) isolates, one with a novel sequence type (^oxf^ST2456) carried the *bla*_NDM-1_ gene, rather than *bla*_OXA-23_, while the other five isolates (^oxf^ST369 and ^oxf^ST2456) did not carry any carbapenem resistance gene. The IC9 (^pas^ST85) isolates (^oxf^ST1089 and ^oxf^ST1936) were the predominant lineage carrying *bla*_NDM-1_, while *bla*_OXA-58_-like genes were present only in singleton/non-IC isolates.

**FIG 4:**
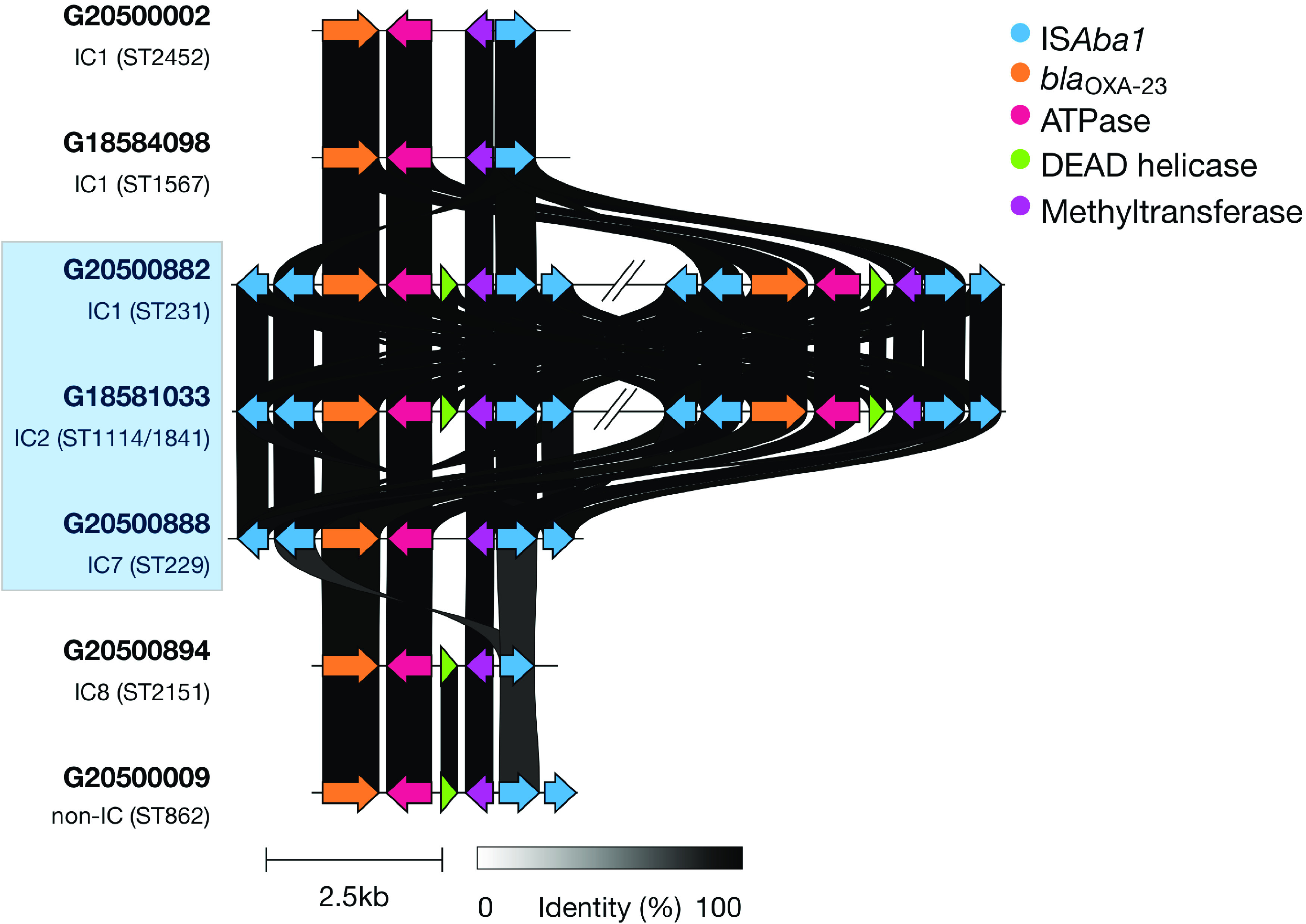
Tn*2006* and Tn*2006*-like transposons carrying the *bla*_OXA-23_ carbapenemase gene in A. baumannii isolates in southwestern Nigeria, 2016 to 2020. A complete IS*Aba1* unit comprises two open reading frames, both indicated with blue arrows. The two Tn*2006* copies in the ^oxf^ST231 (~110 kb apart) and ^oxf^ST1114/1841 (~1.5 Mb apart) isolates are shown. Genetic contexts for lineages highlighted in the light blue box were identified based on annotated complete assemblies while the contexts for unshaded lineages were identified based on identification and annotation of the contig carrying the *bla*_OXA-23_ gene, hence the incomplete repeat element flanks. All STs shown are Oxford STs. Corresponding Institut Pasteur STs are as follows: ^oxf^ST2452, ^pas^ST409; ^oxf^ST1567, ^pas^ST1; ^oxf^ST231, ^pas^ST1; ^oxf^ST1114/1841, ^pas^ST2; ^oxf^ST229, ^pas^ST25; ^oxf^ST2151, ^pas^ST10; ^oxf^ST862, ^pas^ST149.

We generated high-quality complete assemblies for representatives (five isolates) of the carbapenem-resistant lineages (Table S3) and extrapolated the genomic context results/observations to other clonal isolates. All the *bla*_OXA-23_ genes in the different clones in this study had similar immediate genetic contexts; they were all carried on a Tn*2006* transposon flanked by IS*4* family IS*Aba1* inverted repeat elements, or a similar Tn*2006*-like transposon that was missing the truncated DEAD helicase gene (Fig. 4). The nine ^oxf^ST1114/1841 (IC2) isolates carried two distant (~1.5 Mb apart) copies of the *bla*_OXA-23_ gene on the chromosome, each flanked by IS*Aba1*. Interestingly, one of these copies was inserted just upstream of the intrinsic *bla*_OXA-51_-like gene (*bla*_OXA-66_). Among the five ^oxf^ST231 (IC1) isolates, the *bla*_OXA-23_ carbapenem resistance gene was also chromosomally located. Like the ^oxf^ST1114/1841 isolates, these isolates also had two copies of the *bla*_OXA-23_ gene, both proximal, carried on a Tn*2006* transposon, and inserted into the chromosome without being associated with any other mobile elements; one copy was inserted between the *ycfP* and *menH* genes. There was only a single copy of the *bla*_OXA-23_ gene in the seven ^oxf^ST229 (IC7) isolates, which was also present on the chromosome and, interestingly, located within an AbaR4-type genomic island inserted within the *yifB* (*comM* subfamily) gene. No other resistance gene was found in this genomic island.

10.1128/msphere.00098-23.5 TABLE S3 Quality metrics of genome assemblies generated using long reads. Download Table S3, XLSX file, 0.01 MB. Copyright © 2023 Odih et al. 2023 Odih et al. https://creativecommons.org/licenses/by/4.0/ This content is distributed under the terms of the Creative Commons Attribution 4.0 International license.

**FIG 5:**
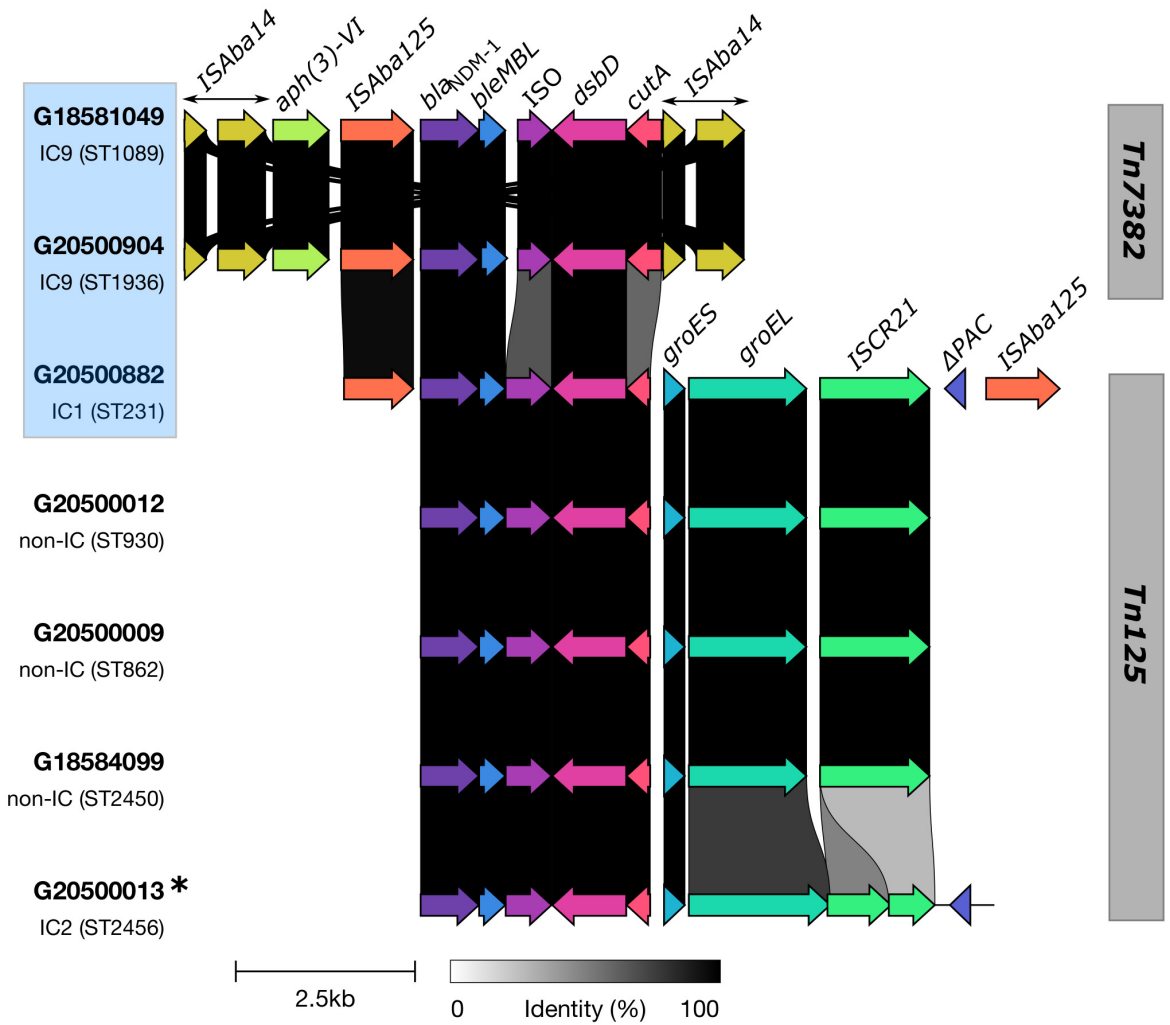
Tn*125* and Tn*7382* transposons carrying the *bla*_NDM-1_ carbapenemase gene in A. baumannii isolates in southwestern Nigeria, 2016 to 2020. Genetic contexts for lineages highlighted in the light blue box were identified based on annotated complete assemblies while the contexts for the other lineages were identified based on identification and annotation of the contig carrying the *bla*_NDM-1_ gene, hence the incomplete repeat element flanks. All STs shown are Oxford STs. Corresponding Institut Pasteur STs are as follows: ^oxf^ST1089, ^pas^ST85; ^oxf^ST1936, ^pas^ST85; ^oxf^ST231, ^pas^ST1; ^oxf^ST930, ^pas^ST32; ^oxf^ST862, ^pas^ST149; ^oxf^ST2450, ^pas^ST1093; ^oxf^ST2456, ^pas^ST2. *, split across two contigs.

As with both copies of the *bla*_OXA-23_ genes carried by the five ^oxf^ST231 (IC1) isolates, the *bla*_NDM-1_ gene was also chromosomally located and was carried on a 10-kb Tn*125* composite transposon flanked by the IS*Aba125* element (Fig. 5). This composite transposon was inserted within a larger 25-kb transposon flanked by an IS*6* family insertion sequence (IS), IS*1006*, which also carried the *aph(6)-Id* and *aph(3″)Ib* genes. The 13 isolates belonging to ^pas^ST85/IC9 (^oxf^ST1089 and ^oxf^ST1936) had a different *bla*_NDM-1_ context. In these isolates, the *bla*_NDM-1_ gene was also located on the chromosome but was associated with an upstream IS*Aba125* element and located within a 7.9-kb Tn*7382* transposon that also carried *aph(3′)-VI* and had two flanking IS*3* family IS*Aba14* direct repeats. The remaining six *bla*_NDM-1_-carrying isolates carried the gene on a Tn*125* composite transposon like the ^oxf^ST231 (IC1) isolates, but we could not determine if this transposon was on the chromosome or a plasmid as there was no representative complete assembly sequenced for this set.

### Plasmids detected in A. baumannii isolates.

Plasmids belonging to 22 distinct replication initiation (Rep) protein types were detected in the isolates (Table S4). Of these, the Rep_3-family plasmids were the most common, with 18 different Rep types belonging to this group detected in the isolates. The remaining four plasmid Rep types detected belonged to the Rep_1 (*n* = 3) and RepPriCT_1 (*n* = 1) groups. The R3-T3 Rep type was the most common among the isolates (*n* = 31 isolates), followed by R3-T1 (*n* = 18), R3-T6 (*n* = 10), and R3-T60 (*n* = 10). Of all Rep types that were present in at least five isolates, none was unique to a specific international clone or ST, and the distribution of these plasmids was not consistent with the phylogeny of the isolates (Fig. S2).

10.1128/msphere.00098-23.2 FIG S2 Plasmid replicons detected in A. baumannii isolates from southwestern Nigeria, 2016 to 2020. Download FIG S2, EPS file, 0.2 MB. Copyright © 2023 Odih et al. 2023 Odih et al. https://creativecommons.org/licenses/by/4.0/ This content is distributed under the terms of the Creative Commons Attribution 4.0 International license.

10.1128/msphere.00098-23.6 TABLE S4 Plasmid replicon types detected in A. baumannii isolates in southwestern Nigeria, 2016 to 2020. Download Table S4, XLSX file, 0.02 MB. Copyright © 2023 Odih et al. 2023 Odih et al. https://creativecommons.org/licenses/by/4.0/ This content is distributed under the terms of the Creative Commons Attribution 4.0 International license.

A large ~111-kb plasmid (pABTJ2__22; GenBank accession no. CP004359.1) belonging to Rep type T3 of the Rep_3 family was the most common plasmid detected in all the isolates (*n* = 21), including the nine ^oxf^ST1114/1841 (IC2) isolates and 12 of the 13 ^pas^ST85 (IC9) isolates. We confirmed the presence of this plasmid and extracted its sequences from the three IC2 and IC9 isolates with complete assemblies. Interestingly, in all the three isolates, none of the pABTJ2__22 plasmids (ranging in size from 112 kb to 116 kb) contained any antimicrobial or disinfectant resistance genes. In the ^oxf^ST1114/1841 strain, this was the only plasmid present; thus, all resistance genes detected in this ST were chromosomally located. The only plasmid-carried resistance gene in the isolates with complete assemblies was the aminoglycoside resistance gene, *ant(2″)-Ia*, which was carried on a small ~6-kb plasmid present in the ^pas^ST85 and ^oxf^ST231 isolates.

## DISCUSSION

Acinetobacter baumannii infections remain a global public health problem, and much remains to be understood about the population structure of this species, especially in understudied settings. In this study, we discovered a large diversity of A. baumannii in the clinical setting of southwestern Nigeria hospitals, with 35 different sequence types, 16 of which were novel. Studies conducted in previously uncharacterized settings have reported similar findings (7, 19). A 2018 retrospective study in Colombia found seven novel STs out of the 11 detected that had been circulating for over 8 years (16). Given the relatively small number of samples in our study, the observed high diversity and lack of phylogeographic clustering may also be indicative of widespread dissemination and underreporting of A. baumannii infections in Nigeria. Clinical microbiology diagnostics remains a challenge in Nigeria and most developing countries. Very few patients are cultured at all, and A. baumannii isolates are particularly difficult to identify using conventional diagnostics (7, 20, 21). The institution of genomic surveillance for AMR in Nigeria has been coupled with efforts to improve basic microbiology capacity, and it is expected that these measures will help plug the existing gaps in the diagnostics and surveillance of these pathogens of major public health importance (22).

This study adds to our understanding that certain clones (ICs 1 to 9) predominate globally and account for a large proportion of the antimicrobial resistance problem in A. baumannii. Nonetheless, we furthermore show that novel and emerging clones are also important in settings of endemicity, as evidenced by the relatively high resistance rates (≥50% resistance to seven of the 12 antimicrobials tested) of these novel STs/noninternational clones, as well as their possession of carbapenem resistance determinants with genetic contexts identical to those present in the international clones. Given that carbapenem resistance contributes to clonal expansion and successful dissemination among A. baumannii strains, the local emergence of a wide variety of carbapenem-resistant variants is a noteworthy trend (15, 23). Moreover, within the known international clones, IC1 (^pas^ST1) and IC2 (^pas^ST2) are still regarded as the most important A. baumannii lineages causing infections and hospital outbreaks globally (3, 24–26). Our study, however, shows an increasing local prevalence of isolates belonging to the recently classified IC9 (^pas^ST85) lineage relative to the globally prevalent IC2 lineage. Even more notable is the associated increased prevalence of the previously rare *bla*_NDM-1_ carbapenem resistance gene. STs belonging to IC9, mostly ^oxf^ST1089, and most of which also carry the *bla*_NDM-1_ gene or other variants such as *bla*_NDM-6_, have been reported more frequently predominantly in Africa and the Middle East (27–33), but also in many other parts of the world (17, 34–36).

Previous studies have highlighted *bla*_OXA-23_ as the predominant carbapenem resistance gene among A. baumannii strains and shown that *bla*_NDM-1_ is rare among A. baumannii strains (37). Our findings, however, show that although OXA-23 carbapenemases are the most common enzymes among A. baumannii in southwestern Nigeria, NDM-1 prevalence is also notably high in our setting and NDM-1 appears to be spreading between different lineages. The Tn*125* composite transposon carrying the *bla*_NDM-1_ gene in the five ^oxf^ST231 (IC1) isolates and six other isolates with distinct STs (^oxf^ST862, ^oxf^ST930, ^oxf^ST2450, and ^oxf^ST2456 [IC2]) is identical in structure and composition to that previously described in an A. baumannii strain isolated in Germany in 2007 and other subsequent studies (37, 38). This *bla*_NDM-1_-carrying Tn*125* transposon, which is believed to have originated in A. baumannii, has also been demonstrated to be very frequently and efficiently mobilized, facilitating its dissemination within and between A. baumannii strains and other *Enterobacterales* (39). BLAST searches of this transposon against the National Center for Biotechnology Information’s nonredundant nucleotide database revealed the presence of this transposon and its derivatives predominantly among A. baumannii and non-*baumannii* Acinetobacter species, but also in different plasmids and chromosomes of E. coli, K. pneumoniae, and other *Enterobacterales*. The *bla*_NDM-1_ among the ^pas^ST85 (IC9) isolates, however, had a different context—a truncated Tn*125* transposon captured within flanking IS*Aba14* direct repeats and recently named Tn*7382* (40). This transposon is only sparsely described in literature but is predominantly found among A. baumannii strains belonging to IC9 (36, 40). Another *bla*_NDM-1_-carrying and Tn*125*-derived transposon, designated Tn*6924*, was recently described in a ^pas^ST85 isolate from Lebanon, indicating the epidemiological importance of this lineage in the increased prevalence and potential dissemination of *bla*_NDM-1_ among A. baumannii strains (41). The increased NDM-1 prevalence in our study and in A. baumannii in general, as facilitated by these highly mobilizable transposons, as well as the potential for intra- and interspecies spread, has notable implications as this enzyme is very potent and has a wider spectrum of hydrolysis for beta-lactams and carbapenems than OXA-23 and OXA-58, thus grossly limiting the already limited number of treatment options for A. baumannii infections (42–45).

Although all the *bla*_OXA-23_ genes in our study had similar contexts, these Tn*2006* and Tn*2006*-like transposon structures were found in diverse chromosomal backgrounds, thus adding to previous knowledge of their rapid and frequent genome mobility (6). One interesting observation was the presence of two copies of the *bla*_OXA-23_ gene in the ^oxf^ST1114/1841 and ^oxf^ST231 strains. A previous study by Zhang and colleagues reported the duplication of a plasmid-borne *bla*_OXA-23_ gene in an A. baumannii strain in the presence of subinhibitory concentrations of carbapenem (46). This duplication was reported to confer improved fitness in carbapenem-containing media but was also associated with a fitness cost in antibiotic-free media. Our findings, however, indicate the maintenance of multiple *bla*_OXA-23_ copies in the chromosomes of multiple strains in distinct lineages, despite the reported fitness costs and resulting instability. This is an important finding as it suggests a key evolutionary adaptation that could lead to the increased potency of OXA-23 carbapenemases and expansion of A. baumannii populations harboring *bla*_OXA-23_, as well as an increased risk of mobilization and onward dissemination of the gene.

This study revealed a diverse array of plasmids, with 22 distinct replication initiation protein types detected in the bacterial isolates and Rep_3 being the most common. The distribution of these plasmids was not consistent with the phylogeny of the isolates, suggesting extensive horizontal exchange between different A. baumannii lineages. The most common plasmid identified, pABTJ2__22, did not contain any antimicrobial or disinfectant resistance genes, despite being present in many of the isolates. In fact, among all the plasmids confirmed in the clones with complete assemblies (^oxf^ST231 [IC1], ^oxf^ST1114/1841 [1C2], ^pas^ST25 [IC7], and ^pas^ST85 [IC9]), only one plasmid carried a resistance gene [*ant(2″)-Ia*]. While this may be surprising given the important role that plasmids are known to play in the dissemination of resistance genes in A. baumannii (3, 47), it is worth noting that the other isolates without complete assemblies could have possessed other resistance genes carried on plasmids that were not confirmed.

Our inability to generate complete assemblies for all the lineages also represents another limitation of this study. As the complete transposon structures were not assembled into single contigs in the strains without complete assemblies, it is not possible to be definitive about the transposon structures of these strains. Nevertheless, the only missing bits in the structures were repeat sequences, which are difficult to assemble with short-read data, thus providing strong evidence for the presented structures. Another limitation of this study was that we did not determine the phenotypic susceptibility of the carbapenem-resistant strains to colistin, which is one of the few remaining antimicrobials with activity against carbapenem-resistant A. baumannii. However, there were no colistin resistance determinants detected in the isolates, suggesting that they may be susceptible to colistin.

### Conclusion.

Acinetobacter baumannii strains in the hospital setting in southwestern Nigeria are highly phylogenetically diverse, are highly resistant to antimicrobials, and may be underreported, indicating the urgent need to improve diagnostic capacity for and surveillance of A. baumannii infections both in Nigeria and in other understudied settings. Our findings also suggest that there is frequent dissemination of carbapenem resistance genes between the different A. baumannii lineages, as well as integration and possible maintenance of these genes in the chromosomes. More local studies are needed to characterize the hospital burden of A. baumannii infection in Nigeria and identify contributors to environmental and clinical spread.

## MATERIALS AND METHODS

### Ethical considerations.

This study was approved by the University of Ibadan/University College Hospital (UI/UCH) Ethics Committee (UI/EC/19/0632). Patients were not actively recruited for this study, and all associated patient data were anonymized before being retrieved for analysis.

### Isolate collection.

All Acinetobacter isolates included in this study were isolated between 2016 and 2020 and submitted to Nigeria’s AMR surveillance reference laboratory at the University College Hospital, Ibadan, Nigeria. These isolates were collected as part of routine surveillance of WHO global priority pathogens in Nigeria and were isolated from blood, cerebrospinal fluid, and rectal swab samples. Submitting laboratories/hospitals included Lagos University Teaching Hospital, Idi-Araba, Lagos State; Clina-Lancet Laboratories, Victoria Island, Lagos State; EL-LAB Medical Diagnostics, Festac, Lagos State; Obafemi Awolowo University Teaching Hospitals Complex, Ile-Ife, Osun State; University College Hospital, Ibadan, Oyo State; University of Ilorin Teaching Hospital, Ilorin, Kwara State; and Babcock University Teaching Hospital, Ilishan-Remo, Ogun State. Where available, associated metadata on sample type, collection date, and patient hospitalization status were obtained from the reference laboratory metadata database. All cryopreserved presumptive Acinetobacter isolates were resuscitated on CHROMagar Acinetobacter medium (CHROMagar, Paris, France) and preliminarily identified using the Vitek 2 automated system (bioMérieux, Inc., Marcy-l’Étoile, France) with Gram-negative identification cards (reference number 21341) according to the manufacturer’s instructions.

### Antimicrobial susceptibility testing.

We determined the phenotypic susceptibility of the isolates to clinically relevant antimicrobials using the Vitek AST N281 cards (reference number 414531) on the Vitek 2 automated system. The following antimicrobials were tested: cefepime, ceftazidime, ciprofloxacin, doripenem, gentamicin, imipenem, levofloxacin, meropenem, minocycline, piperacillin-tazobactam, ticarcillin-clavulanic acid, and tigecycline. The MIC values of all tested antimicrobials except tigecycline were interpreted according to the Clinical and Laboratory Standards Institute (CLSI) clinical breakpoints (48). Tigecycline MIC values >2μg/mL were interpreted as resistant as both the EUCAST guidelines (49) and the CLSI guidelines did not provide clinical cutoffs for tigecycline in A. baumannii. All interpretations were done using the AMR R package (version 1.8.1; https://msberends.github.io/AMR/).

### Genomic DNA extraction and whole-genome sequencing.

We extracted genomic DNA from all the presumptively identified Acinetobacter baumannii complex isolates, prepared double-stranded genomic DNA libraries, and sequenced the libraries on an Illumina platform as previously described (7). After preliminary analyses of the short-read whole-genome sequencing (WGS) data, we selected representatives of the different A. baumannii lineages identified in our data set and carried out long-read whole-genome sequencing of these isolates using the Oxford Nanopore technology to obtain completely assembled genomes for comprehensive analyses. Genomic DNA was reextracted from the selected isolates using the A&A Genomic Mini AX Bacteria+ kit (A&A Biotechnology, Gdańsk, Poland) to obtain less fragmented DNA. Long-read sequencing libraries were then generated using the Rapid Barcoding Sequencing kit (SQK-RBK004) and sequenced on a MinION Flow Cell (R9.4.1) with MinKNOW version 22.08.9 (Oxford Nanopore Technologies, Inc., Oxford, United Kingdom). We then carried out superaccuracy base calling and demultiplexing on the generated reads using Guppy version 6.3.8.

### Whole-genome sequence analysis.

We performed *de novo* genome assembly, species identification, and quality control of all short-read genomes using the Global Health Research Unit (GHRU) protocol (https://www.protocols.io/view/ghru-genomic-surveillance-of-antimicrobial-resista-bpn6mmhe). All assemblies with >300 contigs, genome sizes of <3.3 Mb or >4.7 Mb, an N50 score of <25,000, and containing >5% of contaminating single nucleotide variants of core genes were excluded from downstream analyses. Long-read sequences were assembled using the Trycycler pipeline (50), and the generated circularized assemblies were then polished using Medaka v1.7.2. To generate high-quality complete assemblies, the generated long-read assemblies were then polished with the short reads using Polypolish (51).

We performed a single nucleotide polymorphism (SNP)-based phylogenetic reconstruction analysis to determine the phylogenetic relationships between the identified A. baumannii strains. The raw reads of all samples were mapped to a reference sequence (GenBank accession no. GCA_000830055.1) using the BWA-MEM algorithm with BWA v0.7.17 (52), and possible duplicates were marked and removed using Picard v2.21.6 (http://broadinstitute.github.io/picard). Variant sites were called based on the alignment to the reference sequence using BCFtools v1.9 (53), and low-quality variants were removed. Variant sites were extracted using SNP-sites v2.4.1 (54) and concatenated into pseudogenomes for each of the samples, as well as the reference sequence, after which all pseudogenomes were combined to form a pseudoalignment. We then used RAxML-NG v1.1.0 (55) to construct a maximum likelihood (ML) phylogeny with 50 starting trees and 1,000 bootstrap replicates using the general time reversible gamma (GTR+G) model with the Lewis method for ascertainment bias correction (56).

Multilocus sequence types (MLSTs) were determined from the assembled genomes using the R package MLSTar v0.1.5 (57) with the Oxford and Institut Pasteur MLST schemes (58, 59). The detected sequence types were assigned to IC groups if they had no more than a double locus variation from the Oxford STs in the nine defined ICs (60, 61). Lipooligosaccharide outer core loci and capsular polysaccharide loci were identified using Kaptive v2.0.3 (62). Identified loci with at least a “good” confidence match were reported. All genomes and assembly fragments were annotated using Bakta v1.5.1 (63) with database version 4.0.0. Antimicrobial resistance genes carried by each isolate were identified using AMRFinderPlus v3.10.24 (64) with database version 2022-04-04.1. The intrinsic *bla*_ADC_-family and *bla*_OXA-51_-like genes were excluded from the analysis. Plasmid replicons were identified in the short-read assemblies using the AcinetobacterPlasmidTyping database (65). Only the best-matching replicons (highest percent identity) for each unique contig were reported. Using only the complete assemblies, the genetic contexts of carbapenem resistance genes were observed in Artemis, and genomic resistance islands were identified using the IslandViewer 4 tool (66). Insertion sequence (IS) elements were identified using the BLAST tool on the ISfinder database (https://www-is.biotoul.fr/blast.php). Using GView Server (https://server.gview.ca/), we mapped the draft assemblies of the remaining isolates in each clone to the complete assembly of the long-read sequenced representative strain to determine representativeness. Genomic context results for the representative strain were extrapolated for isolates that had a perfect mapping (>95% coverage) to the representative assembly. For carbapenem-resistant strains without complete assemblies for representative sequences, we identified the contigs carrying the resistance genes and associated elements using BLAST (https://blast.ncbi.nlm.nih.gov/Blast.cgi). Genetic structure comparisons were carried out using Clinker (67).

### Statistical analysis.

All statistical analyses were carried out in R v4.2.1. Proportions were compared between groups using Fisher’s exact test with false-discovery rate correction for multiple testing. Dunn’s test with Bonferroni correction for multiple testing was used following a Kruskal-Wallis test to carry out pairwise comparisons of the numbers of resistance determinants between multiple groups. *P* values less than 0.05 were considered statistically significant.

### Data availability.

The raw reads of all 86 A. baumannii genomes have been deposited in the European Nucleotide Archive (https://www.ebi.ac.uk/ena) with study accession no. PRJEB29739. Accession numbers for each sample are listed in Table S1 in the supplemental material.

10.1128/msphere.00098-23.3 TABLE S1 Accession numbers and epidemiological information of clinical A. baumannii isolates in southwestern Nigeria, 2016 to 2020. Download Table S1, XLSX file, 0.01 MB. Copyright © 2023 Odih et al. 2023 Odih et al. https://creativecommons.org/licenses/by/4.0/ This content is distributed under the terms of the Creative Commons Attribution 4.0 International license.
