# Correction for Odih et al., “High Genetic Diversity of Carbapenem-Resistant *Acinetobacter baumannii* Isolates Recovered in Nigerian Hospitals in 2016 to 2020”

**DOI:** 10.1128/msphere.00659-23

**Published:** 2023-12-21

**Authors:** Erkison Ewomazino Odih, Anderson O. Oaikhena, Anthony Underwood, Yaovi Mahuton Gildas Hounmanou, Oyinlola O. Oduyebo, Abayomi Fadeyi, Aaron O. Aboderin, Veronica O. Ogunleye, Silvia Argimón, Vitus Nnaemeka Akpunonu, Phillip O. Oshun, Abiodun Egwuenu, Tochi J. Okwor, Chikwe Ihekweazu, David M. Aanensen, Anders Dalsgaard, Iruka N. Okeke

## AUTHOR CORRECTION

Volume 8, no. 3, e00098-23, 2023, https://doi.org/10.1128/msphere.00098-23. Page 12, Acknowledgments: The following statement should be added to the end of the first paragraph. “We gratefully acknowledge clinical and diagnostic laboratory staff at the following institutions for contributing material for this study: Babcock University Teaching Hospital, Ilishan-Remo, Ogun State; Clina-Lancet Laboratories, Victoria Island, Lagos State; EL-LAB Medical Diagnostics, Festac, Lagos State; Lagos University Teaching Hospital, Idi-Araba, Lagos State; Obafemi Awolowo University Teaching Hospitals Complex, Ile-Ife, Osun State; University College Hospital, Ibadan, Oyo State; and University of Ilorin Teaching Hospital, Ilorin, Kwara State, Nigeria.”

